# Prioritizing zoonotic diseases utilizing the One Health approach: Jordan's experience

**DOI:** 10.1016/j.onehlt.2021.100262

**Published:** 2021-05-01

**Authors:** Khalid A. Kheirallah, Abdel-Hameed Al-Mistarehi, Lora Alsawalha, Zaidoun Hijazeen, Heba Mahrous, Sami Sheikali, Salam Al-Ramini, Mohammad Maayeh, Rachel Dodeen, Mahmoud Farajeh, Nezar Masadeh, Amer Alemam, Jomana Alsulaiman, Dalia Samhouri

**Affiliations:** a Faculty of Medicine, Jordan University of Science and Technology, Irbid, Jordan; b Jordan Country Office, World Health Organization, Amman, Jordan; c Food and Agriculture Organization of the United Nation, Amman, Jordan; d World Health Organization Regional Office for the Eastern Mediterranean, Cairo, Egypt; e Jordan Ministry of Health, Amman, Jordan; f Jordan Ministry of Agriculture, Amman, Jordan; g Jordan Ministry of Environment, Amman, Jordan; h Yarmouk University School of Medicine, Irbid, Jordan

**Keywords:** One health, Zoonotic diseases, Jordan, Prioritization, Human-animal interface

## Abstract

**Background:**

Zoonotic diseases constitute a threat to humans and animals. The Middle East Region is a hotspot for such a threat; given its geographic location under migratory birds' flight paths, mass gatherings, political conflicts, and refugee crises. Thus, prioritizing zoonotic diseases of national significance is critical for preventing and controlling such threats and optimizing limited resources. Using a multi-sectoral One Health (OH) approach, this study aimed at prioritizing zoonotic diseases of national significance to Jordan and identifying future recommendations and action plans.

**Methods:**

Zoonotic diseases of national significance to Jordan were initially identified (*n* = 27 diseases). In December 2019, national staff from governmental and non-state sectors were invited to develop ranking criteria, including questions and answers choices, and to weigh each criterion. Then, the national staff were asked to assess zoonotic diseases' priority using the developed criteria and provide recommendations and action plans to strengthen multi-sectoral collaboration.

**Results:**

Seven zoonotic diseases were identified as being of great significance. Rabies was ranked as the number one priority disease, followed by middle east respiratory syndrome, avian influenza, brucellosis, leishmaniasis, rickettsiosis, and salmonellosis. The highest weighted criteria used to rank diseases were disease severity, outbreaks profile, and potential human-to-human transmission. Establishing a one-health platform, surveillance, laboratory, preparedness planning, outbreak response, and workforce were suggested as recommendations for approaching the priority diseases. Respondents identified data sharing, coordination, event-based surveillance, and effective communication channels as vital areas to enhance prevention and control strategies, conduct joint outbreak investigations, and improve multi-sectoral collaboration.

**Conclusions:**

This study represents the first attempt to prioritize zoonotic diseases of national significance in Jordan using the OH approach and a semi-qualitative, transparent, and comparative method. Study results can be used as a decision-making guide for policymakers and stakeholders and a cornerstone for combating zoonotic disease threats.

## Highlights

• This is the first regional report to prioritize zoonotic diseases using the One Health (OH) approach.
• A list of country relevant zoonotic diseases was identified, prioritized, and approved using the OH Zoonotic Disease Prioritization (OHZDP) tool.
• Relevant surveillance systems in Jordan should adopt a standardized data sharing mechanism and an event-based method for zoonotic events.

## 1 Introduction

Zoonotic diseases are infectious diseases caused by harmful germs transferred from animals to cause mild to severe illnesses in humans and vice versa [1]. The World Health Organization (WHO) defined “any disease naturally transmissible from vertebrate animals to humans or from humans to animals” as a zoonosis [2]. Most known human infectious diseases originate from animals, and about three-quarters of them are emerging diseases [1,[3], [4], [5]]. Emerging zoonosis is defined as “a zoonosis that is newly recognized or newly evolved, or that has occurred previously but shows an increase in incidence or expansion in geographical, host or vector range” [6]. Domestic animals act as reservoirs for zoonotic agents and transmit pathogens frequently to humans [3,7]. Some zoonotic agents could gradually adapt to human-to-human transmission, as in human tuberculosis. Most of the emerging zoonotic diseases, including avian influenza, Nipah virus infection, Middle East Respiratory Syndrome (MERS), Severe Acute Respiratory Syndrome (SARS), and Swine flu cause severe infections in humans globally, significant public health concerns, and direct human health hazards that led to death [8,9].

Across the globe, the 13 “most common” zoonotic diseases were “most impactful” on poor livestock workers in developing countries and have caused less than 3 billion illnesses and 2.7 million human deaths annually [10]. Human tuberculosis, for example, is considered the second most common cause of death after HIV/AIDS [11], Brucellosis is one of the most common zoonotic diseases causing over 500,000 human cases every year [12], and Rabies, the deadliest zoonotic disease, causes between 30,000 and 70,000 human annual deaths [13]. Besides, the tremendous economic effects of outbreaks and epidemics were estimated to exceed 120 billion dollars for the period between 1995 and 2008 [14].

Late 2019, a novel beta coronavirus, known as severe acute respiratory syndrome coronavirus-2 (SARS-CoV-2), caused one of the deadliest global pandemics in history known as coronavirus disease-19 (COVID-19) pandemic. It resulted in remarkable impacts, was suggested to have a zoonotic origin, and its causing virus (SARS-CoV-2) crossed the animal-human barrier [15]. COVID-19 may be seen as a reminder of the *potential* public health challenges of emerging coronaviruses in line with people and animals' global movements. This is especially true considering the stark global public health challenges associated with the SARS and MERS outbreaks [16,17]. These outbreaks, along with COVID-19, remind us to be “vigilant” and “prepared for the following outbreaks of zoonotic origin” by understanding the human-animal-environment interface's trajectories mitigating similar outbreaks utilizing an integrated approach [18,19]. Thus, to best address zoonotic disease threats, a multi-sectoral One Health (OH) approach is needed.

While zoonosis remains a major global concern, developing countries still at higher risk of such diseases given the nature of contact between animals and humans, limited surveillance capacities, and the limited resources. In this context, countries in the WHO's Eastern Mediterranean Region (EMR) have a unique vulnerability to zoonosis threats [20,21]. EMR is under four of the eight global migratory bird flight paths, [22] [23,24], is vulnerable to emerging infectious and parasitic diseases [25], has been associated with diseases with zoonotic origins (Avian Influenza A, pandemic H1N1/2009 virus, and MERS-CoV) [26,27], and has mass gatherings during Islamic pilgrimage, Hajj, that may increase the risk of disease transmission [[28], [29], [30]]. The Levant, part of the EMR, has witnessed recent political unrests and conflicts that created waves of unprecedented population movements that contributed to the spread of infections and reemergence of infectious diseases [31,32]. Jordan, for example, supports refugees from Syria and other countries who live in camps built quickly over large uninhabited areas. This creates a potentially imbalanced fauna and flora and facilitates human–livestock–wildlife interaction, increasing the risk of zoonotic infections [33]. However, the available healthcare systems are still inadequately prepared to respond to an epidemic effectively [34], and the OH approach is not evident. The OH approach's efforts for prioritizing zoonotic diseases could, then, be critical to equip Jordan to correctly identify and deal with potential epidemics and pandemics in the EMR. This study aimed to prioritize zoonotic diseases of greatest national significance to Jordan using a multi-sectorial OH approach and the OHZDP tool, and to identify future recommendations and action plans.

## 2 Materials and methods

To address zoonotic disease challenges in Jordan, the OHZDP workshop was held in December 2019. The workshop's goal was to prioritize zoonotic diseases of greatest national significance using a standardized multi-sectorial OH approach with equal input from representatives of human, animal (livestock and wildlife), and environmental health sectors, and other relevant partners. National staff from the Ministry of Health (MOH), Ministry of Agriculture (MOA), and Ministry of Environment (MOEnv) served as voting members/core team (*N* = 6 members). A total of 21 members served as advisors/observers to the voting members, while eight served as facilitators to the workshop, including technical officers from World Health Organization. A complete list of involved organizations is provided in Appendix A.

**Fig. 1:**
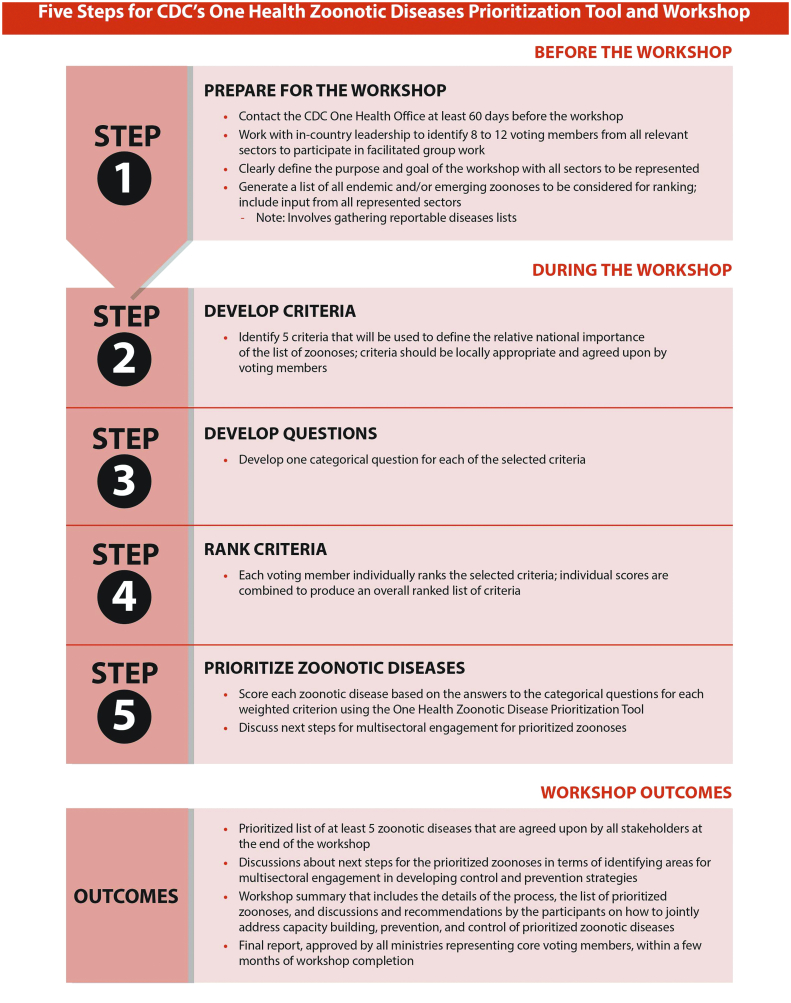
CDC one health prioritization process.

Source: CDC [35].

This OHZDP process used a semi qualitative method developed by the U.S. CDC's OH Office. Fig. 1 represents a description of the used method in detail [35]. The study protocol was reviewed and approved by the Institutional Review Board (IRB) of MOH (IRB approval number is 914/2019). We first formulated the core team, which included representatives from all sectors, as mentioned earlier. The team prepared an initial list of zoonotic diseases (*n* = 33) thought to be of national significance. Afterward, a literature review had been conducted using official national reports, peer-reviewed publications, Gray literature, and Pubmed database. We first reviewed the initial zoonotic disease list and then came up with a final list for prioritization (*n* = 27).

Utilizing group discussion, we asked participants to prepare five criteria to prioritize zoonotic diseases. For each criterion, we then asked them to prepare a question with ordinal answer choices to be used as a scoring system for each question. A higher score indicated a higher propriety of the disease. Each of the above steps was voted upon by the core team.

Voting members then individually ranked their preferences (from zero to 10) for the significance of each criterion. Each voting member's ranking was then recorded into the OHZDP tool associated EXCEL sheets by a facilitator. Then, a group weight for each criterion was estimated as per the OHZDP tool. For each selected zoonotic disease, each question was then answered and a disease score was assigned. Information obtained through literature review as well as the WHO, the World Organization for Animal Health (OIE), and the Program for Monitoring Emerging Diseases (ProMED) was utilized for assigning the scores. Data regarding disease transmission, severity, pandemic and epidemic potential, economic impact, prevention and control, and environmental impact were collected for each zoonotic disease. When information for a zoonotic disease was not reported for Jordan, regional and global data were used. Table 1 contains the ranking criteria, weights, questions, and answers choices.

**Table 1:** One Health zoonotic prioritization tool developed in Jordan.

Rank	Criteria	Weight	Question and its description	Answers	Scores
1	Severity of disease	0.4	Is the disease severe in humans and animals?	A. None B. In animals C. In humans D. Both	A. Score = 0 B. Score = 1 C. Score = 2 D. Score = 3
			Severity is determined by case fatality. Severe: when case fatality, or abortion, is more than 5% in animals or when, in humans, one case fatality.		
2	Epidemiological profile (Incidence and Prevalence)	0.22	Has the zoonotic disease caused any outbreak in humans in the last ten years?	A. No B. Yes	A. Score = 0 B. Score = 1
			Definition of the outbreak: any increase in the number of cases above the expected case count in Jordan. No, the case count does not exceed the normally expected cases in Jordan Yes, there is an increase in the number of cases above the expected cases in Jordan.		
3	Potential transmission (pandemic potentiality)	0.17	Does the disease have the capability of transmission from human-to-human?	A. Never: no reported cases B. Rare: few reported cases C. Sustained: continuously reported cases	A. Score = 0 B. Score = 1 C. Score = 2
			The disease has the capability of transmission from human to human either directly or indirectly, and the answer relies on reported cases. Human-to-Human means: all modes of transmission except induced-transmission (blood transfusion, needle stick, and organ transplant).		
4	Availability of Intervention	0.13	Does the zoonotic disease have control and prevention measures for intervention?	A. None of the measures available B. Some of the measures available C. Most measures available D. All measures available	A. Score = 0 B. Score = 1 C. Score = 2 D. Score = 3
			Measures are diagnostic capacities, vaccination, surveillance, rapid response team, and risk communication. Available measures do not take higher priority. Some: one or two measures Most: 3 to 4 All: all measures available		
5	Socio-economic-environmental impact	0.08	Does the disease affect the production, trade, and movement of animals and humans?	A. None B. Only humans C. Only animals D. Both (humans and animals).	A. Score = 0 B. Score = 1 C. Score = 2 D. Score = 3
			None: no effect. Only animal: decrease animal production and trade, and costly treatment and vaccination. Only human: decrease in human productivity; affect tourism, costly treatment, and vaccination. Animal and human: both effects as above.		

Following score assignment, a “decision tree analysis”, as provided by the OHZDP was utilized for ranking the list national zoonotic diseases. Each *weighted* criterion was recorded into the OHZDP tool to provide a weighted score for each disease. The weighted scores for all criteria, questions, were then summed and normalized in the OHZDP tool to provide a score of 1 or less. The disease with the highest score value had the highest priority.

Zoonotic diseases' raw and normalized scores were then discussed with participants for approval and voting. The final approved list of ranked diseases was further considered for next steps and action plans to address threat related to zoonotic diseases of national significance.

### 2.1 Development of OHZDP criteria

The criteria identified by participants to rank the zoonotic diseases of national significance are provided in order of importance in Table 1, with details in Appendix B. These included:

1. Severity of the zoonotic disease.
2. Epidemiological profile (Incidence and Prevalence).
3. Potential transmission (pandemic potentiality).
4. Availability of Intervention.
5. Socio-economic-environmental impact.

## 3 Results

The initial zoonotic disease priority list included 27 zoonotic diseases and was created based on the reports provided by official (governmental) publications, peer-reviewed publications, Gray literature, and PubMed database. These diseases were scored by participants using our developed prioritizing criteria. Table 2 presents the raw and normalized scores for zoonotic diseases of national significance considered for prioritization. We reached a final priority list that included seven diseases (Table 4, Table 5). The final list that was voted up on included Rabies, MERS-CoV, zoonotic avian influenza, brucellosis, leishmaniasis, rickettsiosis, and Salmonellosis. (See Table 3.)

**Table 2:** Jordan's developed list of priority zoonotic diseases.

#	Disease	Raw score	Normalized final score
1	Rabies	0.83	1.00
2	Middle East Respiratory Syndrome- coronavirus (MERS-CoV)	0.78	0.94
3	Salmonellosis	0.68	0.82
4	Zoonotic avian influenza	0.65	0.78
5	Leishmaniasis	0.57	0.69
6	Rickettsiosis	0.53	0.64
7	Brucellosis	0.52	0.63
8	Shigellosis	0.42	0.51
9	*Escherichia coli*	0.40	0.49
10	Malaria	0.39	0.48
11	Tuberculosis	0.36	0.44
12	Anthrax	0.32	0.38
13	Toxoplasmosis	0.32	0.38
14	Leptospirosis	0.19	0.23
15	Q fever	0.18	0.21
16	Botulism	0.13	0.16
17	Plague	0.13	0.16
18	Echinococcosis	0.11	0.14
19	Dengue Fever	0.09	0.10
20	West Nile Fever	0.09	0.10
21	Sarcoptic mange	0.09	0.10
22	Glanders	0.04	0.05
23	Rift Valley Fever	0.04	0.05
24	Tick-borne relapsing fever	0.00	0.00
25	Orf (contagious pustular dermatitis)	0.00	0.00
26	Babesiosis	0.00	0.00
27	Dermatophytosis	0.00	0.00

**Table 4:** Final zoonotic diseases selected in Jordan.

Rank	Zoonotic disease	Justification
1	Rabies	Same as the prioritized list
2	MERS-CoV	Same as the prioritized list
3	Zoonotic avian influenza	Voting members agreement
4	Brucellosis	Voting members agreement
5	Leishmaniasis	Same as the prioritized list
6	Rickettsiosis	Same as the prioritized list
7	Salmonellosis	Voting members agreement

**Table 5:** Suggested actions to develop strategies against zoonotic diseases.

Proposed activities	Ministries involved	Partners
Theme 1: Standardized data sharing mechanism		
Establish a National One Health committee with specific terms of reference (ToR) and standardized operational procedures (SOPs) to review National legislation to facilitate the implementation of IHR in the animal health sector	MOA/ MOH & All relevant sectors	Nationals to complete
Consultation meeting to discuss the development of electronic information sharing platform data sharing between surveillance in both animal and public health sectors	MOA/MOH	WHO/FAO/OIE
Training personnel on animal disease data reporting	MOA/MOH	WHO/FAO/OIE
Conduct regular meeting between private and public sectors to expand the reporting sources to private sectors	MOA/MOH	Nationals to complete
Meeting with relevant stakeholders to develop joint surveillance system SOPs	MOA/MOH	Nationals to complete
Prepare Legal framework for Zoonotic diseases reporting	MOA/MOH/ Ministry of Justice	Nationals to complete
Multisector meeting to develop event-based surveillance system and/or syndromic platform	MOA/MOH & other relevant sectors	Nationals to complete
Development of subnational (Governorates) strategies and operational plan for Zoonosis	MOA/MOH & other relevant sectors	Nationals to complete
Review of subnational legislation, policies, rules, and administrative arrangements in light of revised national policy and legislation.	MOA/MOH & other relevant sectors	Nationals to complete
Conducting a training needs assessment for both sectors (Human and Animals)	MOH/ MOA	WHO/OIE/FAO/JUST
Develop and conduct Continuous Professional Training	MOH/ MOA	WHO/OIE/FAO/JUST
Develop and implement short in-service and refresher training modules on zoonotic diseases (surveillance, lab diagnosis sample shipment, etc.) for health & non-health professionals	MOH/ MOA	WHO/OIE/FAO/JUST
Reviewing the existing training modules/plans and developing/implementing comprehensive in-service and refresher courses/modules training modules on surveillance, lab diagnosis, and sample shipment for the field and lab persons	MOH/ MOA	WHO/OIE/FAO/JUST

Theme 2: Event-based surveillance systems and communication channels for zoonotic events		
Preparing national Zoonotic Disease Plan for zoonotic diseases	MOA/ MOH	One Health Committee
Enhance communication between sectors	MOA/ MOH	One Health Committee
Animal health legislations updating	MOA	National Authorities
Jointly analysis of the zoonotic diseases data for appropriate planning of joined response.	MOA/ MOH	MOH/MOA/WHO/FAO/OIE
Include one health concept in teaching and training curricula for medical and veterinary sciences	MOA/ MOH/ JUST	JUST
Advocacy and awareness sessions to be implemented for public and private professionals (health/non-health) for reporting of zoonotic pathogens for better control measures	MOA/ MOH	WHO/ FAO
Implementation of communication plans developed for risk communication to the general population on prevention/reporting of zoonotic diseases	MOA/ MOH	WHO/ FAO

Abbreviations: ToR: Terms of Reference; SOPs: Standardized Operational Procedures; IHR: International Health Regulations; MOA: Ministry of Agriculture; MOH: Ministry of Health; WHO: World Health Organization; FAO: Food and Agriculture Organization of the United Nations; OIE: World Organization for Animal Health; JUST: Jordan University of Science and Technology.

After finalizing the list, participants discussed recommendations, next steps, and action plans to address the top ranked (priority) diseases using a multi-sectorial OH approach. Participants were first asked to suggest general recommendations for approaching the priority diseases without considering their respective institutions' constraints. A summary of the most prominent recommendations organized by them included a OH platform, surveillance, laboratory, preparedness planning, outbreak response, and workforce. After that, more specific recommendations for each theme were built.

1. One Health platform

To identify a OH platform, the roles and responsibilities of different stakeholders should be clearly stated. A standardized operations procedure for communication and collaboration between relevant sectors of MOH, MOA, and MOEnv should also be established. A clear methodology for regular monthly exchange of reports within each sector and quarterly exchange of reports between different sectors should be developed. A series of simulation exercises to evaluate the national preparedness and response capacities for priority public health and zoonotic diseases of national and international significance was suggested.

2. Surveillance

The team suggested establishing clear guidelines for case definitions and a joint surveillance system regarding the seven priority zoonotic diseases. The notification process for zoonotic diseases should be enhanced to identify and respond to potential outbreaks swiftly.

3. Laboratory

A multi-sector task force to reform and consolidate all national committees into a single, multi-sector national committee should be integrated along with improving peripheral labs' capacity. A data, samples, and sharing platform should be developed among different labs in all sectors.

4. Preparedness planning

Joint risk assessment activities for the seven prioritized zoonotic diseases should be regularly conducted along with a clear plan for the OH committee to enhance timely information sharing.

5. Outbreak response

Capacity-building for the joint Rapid Response Teams (RRT) should be established, and standardized operational plans for proper investigation and rapid response to potential zoonotic diseases outbreak were suggested. These activities should include regular national Simulation exercises for RRT.

6. Workforce

A Field Epidemiological Training Program for Veterinary medicine should be established along with a capacity-building strategy for public health sectors.

### 3.1 Suggested next steps

Finally, the ministries involved in formulating policies regarding zoonotic diseases of national significance and the organizations observing the process were allowed to suggest next steps to fine-tune multi-sectoral capacity building in surveillance and laboratory, prevention and control plans, and conduct joint outbreak activities. Table 5 summarized the suggested next steps under two main themes:

(1) Development of a standardized data sharing mechanism between animal health and public health surveillance systems; and.
(2) Development of event-based surveillance systems and communication channels for zoonotic events.

## 4 Discussion

The OH considers the “human-animal-environmental interdependence” through “a multi-sectoral, collaborative, and trans-disciplinary” approach working at the local, national, regional, and global levels [36,37]. This approach could provide effective zoonotic diseases' prevention and control programs, including broader socio-economic and ecological determinants of health [18,38]. The CDC established the first OH Office in 2009 after the Avian Influenza Crisis [39]. As such, zoonotic diseases of significance should be jointly addressed by multi-sectoral sectors, especially after considering that zoonoses account for approximately 60% of all emerging infectious diseases [4,40,41]. Experts from CDC's OH Office lead One Health Zoonotic Disease Prioritization (OHZDP) workshops in countries to help prioritize zoonotic diseases of national concerns [42].

OHZDP workshops were held to collaborate between representatives of human, animal, and environmental health sectors with a clear objective; to prioritize zoonotic diseases on a national level. Collaboration across these multi-sectors would decrease the demand for scarce resources and establish a successful joint response that could effectively mitigate outbreak risks, implement disease control strategies, and identify future recommendations and action plans. Prioritizing zoonotic diseases using multi-sectoral collaboration is of utmost importance to establish sustained, proactive, and routine partnerships. As such, joint prioritization of zoonotic diseases is expected to positively reflect a well-organized surveillance, develop laboratory capacity, target active outbreak prediction, implement common disease control activities, and identify joint research activities utilizing all sectors [43].

In Jordan, the OHZDP workshop's overarching objectives were to strengthen multi-sectoral collaborations by mutually identifying a list of priority zoonotic diseases and to identify a clear road map to better deal with potential zoonotic disease outbreaks. The workshop's timing came while the global was preparing for the COVID-19 pandemic, which sent a clear message of the importance of implementing clear steps to deal with potential zoonotic diseases. Therefore, Jordan's prioritization process is a cornerstone that will reflect on the region as a whole. The OH approach is first addressed not only by the prioritization process, but also by bringing the multi-sectoral team into one table where decisions are mutual and inclusive for national responses.

The workshop identified gaps in disease detection, surveillance, and reporting between the health and animal sectors. While the MOH's surveillance systems were well established, the MOA utilized an outdated system that needs updating. A collaborative platform for OH suggested integration surveillance and detection that would benefit all stakeholders. Until recently, information sharing among animal and health sectors in the event of zoonotic outbreaks was on a case-by-case basis without a well-established coordination mechanism. As well, the OH approach was not fully functional, and the notification system was not coordinated. These challenges are still a major limiting factor for detecting and preventing the emergence of a Public Health Emergency of International Concern (PHEIC) through real-time surveillance. In view of the above, the WHO-EMR office provided support to countries, including Jordan, to identify and run the different systems, mechanisms, and practices to better address and respond to emerging and re-emerging zoonotic diseases. Despite traditional challenges in low-income countries [44], Jordan has already established itself among EMR countries where prioritization of zoonotic diseases is now available, and the OH approach is ready for the next step.

The current COVID-19 pandemic is a reminder of the potential zoonotic disease's role in public health and highlights the need for globally operationalizing the OH approach. The limited resources in developing countries are also a cue of the crucial need for implementing a global OH approach in low-resource settings. Within this context, the OHZDP workshop in Jordan is a prime example of the country's intentions to initiate the OH approach. The current workshop's activities could then be seen as an active commitment of stakeholders to be a regional role model. Today, Jordan will have a standardized list of such diseases that will better mitigate potential epidemics. Without this list, the efforts to combat zoonosis will be out of focus and uni-sectoral. On the other hand, the successful completion of this task depended on mutual understanding, transparency, equal representation, and agreement from all stakeholders. The country's ownership of the process gives the prioritized list an official entity that is much needed for future steps. Instead of having multiple lists of zoonotic diseases, one list is now sufficient to be representative to all stakeholders.

In the current study, the derived disease criteria scores were not only “rational” but also consistent with other studies presenting similar criteria [45]. Comparing our findings to other countries [[46], [47], [48], [49], [50]], the highest criterion was “severity of disease in humans” in all prioritization workshops, which indicates “strength and robustness” of the process of the OHZDP tool. This is despite the flexible nature of the used OHZDP tool. Disease impact, epidemic potential, and transmission were also reported [[46], [47], [48], [49], [50]]. However, the ownership of the list by stakeholders still makes it unique to Jordan. This is one of the strengths of the used tool. Further, the next step actions established were extremely relevant to improving global health security. This includes enhancing data sharing and improving communication between ministries, strengthening the OH workforce. Identification of priority action items will also empower stakeholders in Jordan to solicit or engage funding partners.

This study has few limitations. First, the workshops were conducted in December 2019 before evolving the COVID-19 pandemic affected our region, and because of the unknown source of this disease at that time, COVID-19 was not included in our list of diseases. Second, there is a lack of national-level data regarding zoonotic disease, especially from the MoEnv, and, to a lesser degree, the MOA. This may have biased the list towards the MOH side where data is up-to-data. However, we tried to overcome this limitation by engaging experts from non-state actors, academicians, and WHO to reflect on regional and global data. Still, lack of data highlighted critical areas for upcoming partnership and demonstrated needs for enhanced surveillance. Although there may be differing perceptions regarding the validity of prioritization, the exercise's importance should rest on its transparency and determine the relative position each disease occupies compared to others, irrespective of methods used [[51], [52], [53], [54]].

## 5 Conclusion

Utilizing the CDC OHZDP tool, a list of priority zoonotic diseases was successfully established in Jordan as a cornerstone for the next steps towards a One Health approach. Better multi-sectoral planning, communication, and collaboration between humans, animals, and the environment sectors have been established. This will improve coordination, mobilization, and early detection, reporting, and control of zoonotic diseases and other health threats. This advancement of the One Health approach in Jordan will make a significant difference in improving livelihoods and the health of people, animals, and the environment.

## Compliance with ethical standards

All conducted procedures in this study involving human participants were reviewed and ethically approved by the Institutional Review Board (IRB) of the Ministry of Health with an IRB approval number of 914/2019.

## Availability of data and materials

The database generated and analyzed during the current study is available with the corresponding author.

## Funding

This research did not receive any specific grant from funding agencies in the public, commercial, or not-for-profit sectors.

## Author statement

The author have read and approved the revised version submitted.

## Authors' contributions

Khalid A. Kheirallah and Lora Alsawalha: conceptualization, methods, supervision, validation, and writing the original draft. Abdel-Hameed Al-Mistarehia, Zaidoun Hijazeen, Dalia Samhouri and Heba Mahrous: formal analysis, visualization, and review and editing the manuscript. Sami Sheikali, Salam Al-Ramini, Mohammad Maayeh, Rachel Dodeen, Mahmoud Farajeh and Nezar Masadeh: investigation, review and editing the manuscript, and analysis. Amer Alemam and Jomana Alsulaiman: reviewing and editing the manuscript and data visualization. All authors have approved the content, fulfill the authors' criteria, and have contributed significantly to work. All authors presented substantial contributions to this study and participated in the submitted version's correction and final approval.

## Declaration of Competing Interest

The authors declare that they have no financial and/or competing interests.

## Figures and Tables

**Table:** 

Name	Number of members
Ministry of Health (MOH),	14
Ministry of Agriculture (MOA),	9
Ministry of Environment (MOEnv),	2
Jordan University of Science and Technology (JUST),	1
World Health Organization (WHO),	3
Food and Agricultural Organization of United Nations (FAO),	1
Jordan Civil Aviation Regulatory Commission (CARC),	1
Eastern Mediterranean Public Health Network and Global Health Development (EMPHNET/GHD),	1
International Organization for Migration (IOM),	1
Jordan Food and Drug Administration (JFDA),	1

**Table 3:** Priority zoonotic diseases selected in Jordan during the One Health Zoonotic Disease Prioritization workshop.

Zoonotic disease	Causative agent	Human disease burden	Animal disease burden	Diagnostics, treatment, and prevention
Rabies	Virus	According to MOH, 4753 patients were treated for rabies exposure in 2013, but no human rabies cases were reported for the last three years. In Jordan, between 2000 and 2007, a total of 15,690 animal bites were reported averaging 1961 annual cases (minimum 1332 in 2002 – maximum 2921 in 2007).	MOA reported a total of seven cases and seven deaths to OIE in 2013. According to MOA reports, 22 cases were documented and reported in Jordan in 2018. In the MENA, dogs are the main reservoir for rabies, and it affects more domestic carnivores (50% of cases) than farm animals (40% of cases).	An effective animal vaccine exists, and human vaccines are available. Post-exposure prophylaxis is available. Treatment is supportive.
Middle East Respiratory Syndrome (MERS)	Virus	Out of the 27 total cases of MERS-CoV in Jordan, 7 cases died. In 2019, 13 laboratory-confirmed cases were reported to be linked to an outbreak in Saudi Arabia in April 2019. As of January 2019, 2298 laboratory-confirmed human cases were identified from 27 countries with 811 deaths (fatality rate = of 35.2%). In the Middle East region, the affected countries with primary cases include Saudi Arabia, Qatar, Jordan, UAE, Oman, Kuwait, Egypt, Yemen, Lebanon, and Iran in Middle East.	In Jordan, in 2016, 28 positive MERS-CoV camel samples seroprevalence was 78% less one year, 69% 1 to 2 years, and 100% over two years. In 2019, 11 PCR positive samples and 2 out of 12 seroprevalences with seropositivity. In Saudi Arabia, seventy-five dromedary camels (*N* = 584) were positive for MERS-CoV. Anti-MERS ELISA assays showed that 70.9% of camels related to human cases had antibodies to MERS-CoV.	No vaccine exists. Treatment with supportive care.
Zoonotic avian influenza	Virus	An upsurge of influenza activity during 2014/15, 2015/16, and 2017/18 seasons was also reported.	Jordan MOH report published in 2017 reported that on March 23, 2006, Jordan reported an outbreak of HPAI virus, type H5N1, in poultry. This was the first confirmed occurrence of HPAI in Jordan. Several of Jordan's neighboring countries also announced outbreaks of the H5N1 virus in poultry.	Treatment with oseltamivir and supportive care.
Brucellosis	Bacteria	According to the MOH report, fifty-five cases were reported in Jordan in the first six months of 2019. In Jordan, the number of human Brucelloses ranged between 132 cases in 2005 and 273 cases in 2015, with a total of 1554 cases (between 2005 and 2014). The disease is endemic in Jordan. The incidence rates in the last five years range from 3.6 to 6.6 per 100,000 population with a median of 5.6 per 100,000. A total of 23.4 infections diagnosed per millions of inhabitants were reported in Jordan, while in Syria and Iraq, the number of diagnosed cases per million inhabitants were 1603.4 and 278.4, respectively. Brucellosis burden is considered the highest reported in North Africa and Middle East (up to 269 cases per 100,000 person-years).	Among animals in Jordan, 53 positive animal cases were reported by MOA 2018, with prevalence estimates, in 2009, in cattle (*N* = 671, 10.1%), in sheep (*N* = 602, 14.3%), and in goat (*N* = 1100, 27.7%). No estimates were provided for camels or buffalos. A similar number of reported cases among animals was reported in 2019.	A vaccine is available for animals and treatment with antibiotics is available for humans.
Leishmaniasis	Parasite	The average incidence of CL (2009–2018) was 2.2 per 100,000, with small outbreaks of focal nature frequently occurred during the last ten years. One LV case was reported in 2019 from Wadi-Araba. The disease is sporadic, with about 23 cases reported from 1962 up to Oct. 2019, 5 cases of which were imported. Two deaths in 2003 and one death of an imported case in 2018 due to late diagnosis. CL is endemic in Jordan, especially in the Jordan valley. Outbreaks of CL have been reported in Aqaba, North Agwar, and South Shuneh. However, there has been severe underreporting of the cases by an estimated factor of 47 times. A study conducted in 2019 found that 20 and 9 out of the inspected 66 patients (39 Jordanian and 27 Syrian) were infected with L. *major* and L. *tropica*, respectively. A total of 558 Syrian refugee patients were clinically diagnosed with CL during 2010–2016. L. Major is the standard and more widespread form of cutaneous leishmaniasis in Jordan.	No reported cases in the last five years. Diagnostic tests are not available in Jordan.	No vaccination is available. Treatment is available for humans.
Rickettsiosis	Bacteria	Epidemiological patterns of Mediterranean Spotted Fever (MSF) in Southern Jordan children were presented between 2013 and 2015. A total of 35 male and 20 female patients (age mean (SD) = 6 ± 3.6) were identified. The incidence of MSF was 7.9 cases/100,000 inhabitants/year; MSF affected 89% of individuals in the summer, 74.5% of those living in a rural area with tent housing, and 100% of those who had contact with animals.	No cases were reported in the last five years.	Treatment is available for humans.
Salmonellosis	Bacteria	Between 2005 and 2014, out of five MOH sites, 2 to 8 per 1000 specimens yielded Salmonella from 10,000–20,000 specimens annually.	One case was reported in 2019, and one case in 2018. The prevalence estimates of *Salmonella enterica* were: 1.6% in bulk tank milk and 3.8% in fecal samples.	Treatment is available for humans.

Abbreviations: MOH: Ministry of Health; MOA: Ministry of Agriculture; OIE: World Organization for Animal Health; MENA: Middle East and North Africa; MERS-CoV: Middle East Respiratory Syndrome- coronavirus; WHO: World Health Organization; PCR: Polymerase chain reaction; ELISA: Enzyme-Linked Immunosorbent Assay; HPAI: Highly Pathogenic Avian Influenza; CL: Cutaneous Leishmaniasis; VL: Visceral Leishmaniasis; L. Major: Leishmaniasis Major; L. Tropica: Leishmaniasis Tropica; MSF: Mediterranean Spotted Fever.

## Appendix B Development of One Health zoonotic prioritization criteria for ranking zoonotic diseases in Jordan

1. Severity of the zoonotic disease

The severity of the zoonotic disease criterion was determined by case fatality using the question as follows: “Is the disease severe in humans and animals?”. In humans, one case fatality qualifies the disease as severe, while in animals the disease was considered severe if case fatality, or abortion, is more than 5%. Zoonotic diseases were classified as not severe (scored as 0), severe in animals only (scored as 1), severe in humans only (scored as 2), or severe in animals and humans (scored as 3).

2. Epidemiological profile (incidence and prevalence)

This criterion was defined by the occurrence of outbreaks within humans in the last ten years in Jordan utilizing the “Has the zoonotic disease caused any outbreak in humans in the last ten years?” question. An outbreak was determined to occur if there was an increase in the number of cases above the expected case count in Jordan. Accordingly, when no outbreak was determined, the disease was scored as 0, but if an outbreak happened among humans in Jordan, it was scored as 1.

3. Potential transmission (pandemic potentiality)

Potential transmission (pandemic potentiality) was defined as the disease's capability to transmit from human-to-human, and it was determined by the question: “Does the disease have the capability of transmission from human-to-human?”. Human-to-human transmission included all transmission modes either directly or indirectly except induced-transmission (blood transfusion, needle stick, and organ transplant). The answer/scoring for this criterion relied on the number of reported human-to-human transmission cases. When the disease did not have any reported human-to-human transmission, the answer was “Never” and the score was 0. When the human-to-human transmission was found in “Few reported cases” the answer was “Rare” and the disease scored “1”. When continuous reported cases were noted, the potential human-to-human transmission was reported as “Sustained” and the disease scored “2”.

4. Availability of intervention

Availability of intervention criterion was measured using the following question; “Does the zoonotic disease have control and prevention measures?”. The criterion assessed the availability of diagnostic capacities, vaccination, surveillance, rapid response team(s), and risk communication strategies in Jordan. Available measures were noted to take higher priority (score). When “None of the measures are available”, the disease scored 0. When one or two measures were reported, the response was “some of the measures are available” and the disease scored 1. When three to four measured were reported, the response was “most measures are available” and the disease scored 2. When “all measures are available”, the disease scored 3.

5. Socio-economic-environmental impact

This criterion assessed the impact of the disease in three dimensions (Socio, economic, and environmental) utilizing the following question; “Does the disease affect the production, trade, and movement of animals and/or humans?”. When no effect was noted, the response was reported as “None”, or no effect, and the disease was coded as 0. When the effect was noted among humans only, “Decrease in human productivity, affect tourism, and costly treatment and vaccination”, the disease scored 1. When the disease effect was noted among animals only, “decrease animal production and trade, and costly treatment and vaccination”, the disease scored 2. When the effect was noted among both humans and animals, the disease scored 3.
